# Immune checkpoint LAG-3 governs stage-dependent and disease-associated microglial modules in ALS model mice

**DOI:** 10.1186/s12974-026-03919-8

**Published:** 2026-06-25

**Authors:** Yuta Morisaki, Nanaka Nomura, Motoki Ohshima, Miruto Matsuda, Okiru Komine, Takashi Okuda, Koji Yamanaka, Hidemi Misawa

**Affiliations:** 1 Division of Pharmacology, Faculty of Pharmacy, Keio University, Tokyo, Japan; 2 Department of Neuroscience and Pathobiology, Research Institute of Environmental Medicine, Nagoya University, Nagoya, Japan

**Keywords:** Amyotrophic lateral sclerosis, LAG-3, Microglia, Disease-associated microglia, Immune checkpoint, Neuroinflammation

## Abstract

Immune checkpoint molecules, inhibitory receptors originally characterized in T cell biology, have recently emerged as regulators of microglial function in neurodegeneration, yet their roles in amyotrophic lateral sclerosis (ALS) remain unexplored. Here, we investigated LAG-3, an inhibitory immune checkpoint receptor, in microglial regulation during ALS pathogenesis using SOD1^G93A^ mice. LAG-3 expression was progressively upregulated in spinal cord microglia during disease progression, and LAG-3-high microglia exhibited a disease-associated microglia (DAM) transcriptional signature. Genetic deletion of LAG-3 produced a biphasic phenotype, with accelerated disease onset but significantly prolonged disease duration. LAG-3 deficiency enhanced inflammatory microglial responses at the early disease stage, whereas at the late stage it suppressed inflammatory signaling while selectively preserving phagocytic effector gene expression, demonstrating that LAG-3 dissociates the inflammatory and phagocytic modules within the DAM program in a stage-dependent manner. These transcriptional changes translated into enhanced phagocytic capacity in primary microglia and amelioration of the spinal cord environment through suppression of inflammatory pathways and restoration of oxidative phosphorylation. Our findings identify LAG-3 as a stage-dependent regulator of microglial functional states in ALS and support the concept that immune checkpoint molecules constitute a class of module-level regulators of microglial function in neurodegeneration.

**Graphical Abstract:**

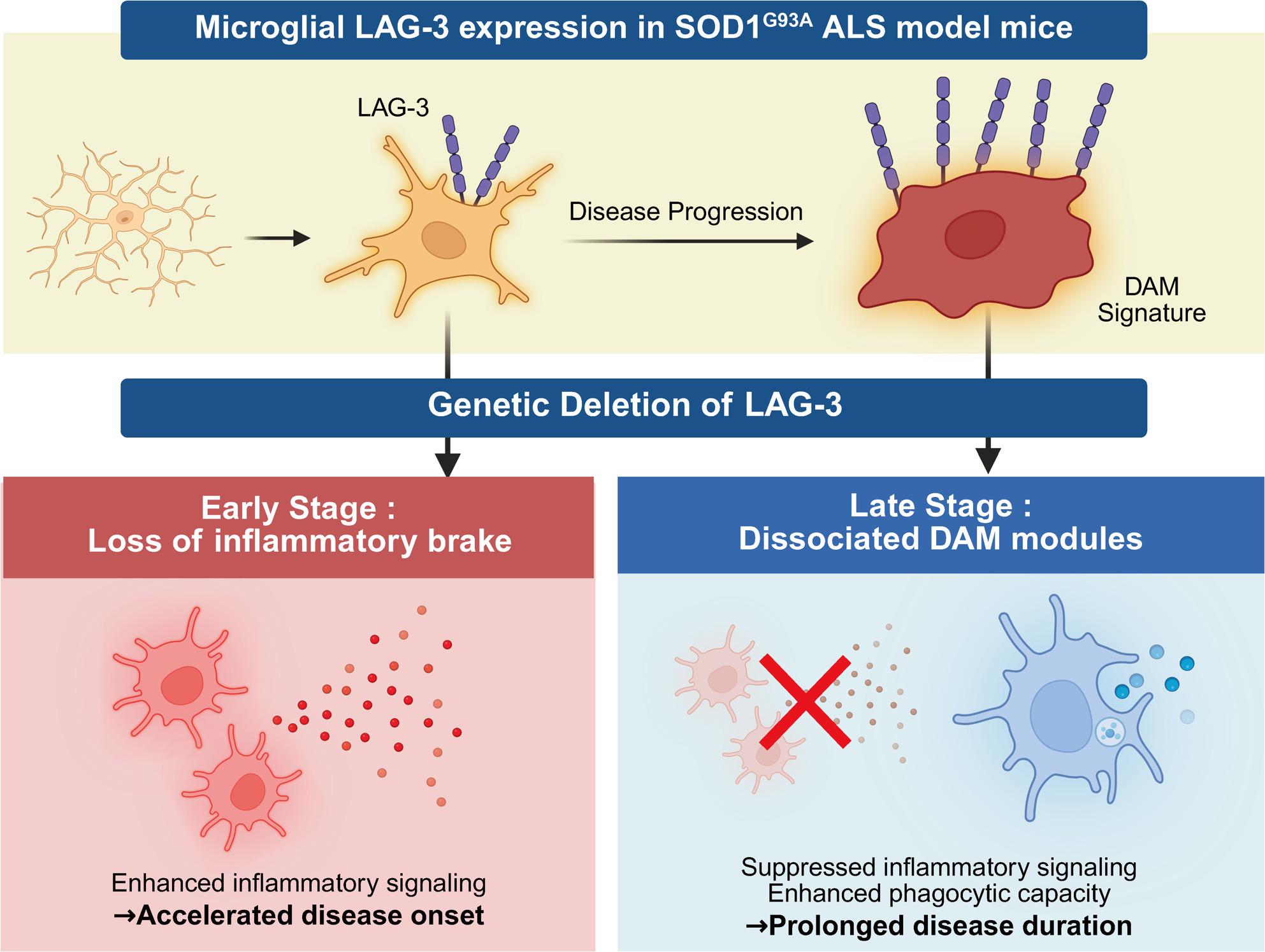

**Supplementary Information:**

The online version contains supplementary material available at 10.1186/s12974-026-03919-8.

## Background

Amyotrophic lateral sclerosis (ALS) is a progressive neurodegenerative disease characterized by the selective loss of motor neurons [1, 2]. Beyond motor neuron-intrinsic mechanisms, neuroinflammation mediated by glial cells has been recognized as a critical determinant of disease progression [3–5]. Among the cellular mediators of neuroinflammation, microglia play a pivotal role in shaping the disease course [6–8]. Microglial responses evolve dynamically as the disease progresses; they initially exhibit a neuroprotective phenotype but gradually acquire a dysfunctional profile characterized by chronic inflammation and loss of homeostatic capacity [9–12]. However, the molecular mechanisms that govern these stage-dependent functional transitions remain to be fully elucidated.

Advances in single-cell transcriptomics have revealed that microglia in neurodegenerative conditions adopt a conserved activation state known as disease-associated microglia (DAM), characterized by the upregulation of genes related to lipid metabolism and phagocytosis and the concurrent loss of homeostatic markers [13, 14]. This transcriptional signature has since been documented in ALS, in both mouse models and human patient tissues [15–18]. The DAM program, however, is not a monolithic response; it includes functionally distinct modules, such as phagocytosis, lipid handling, and inflammatory signaling, that may be independently regulated [19, 20]. Elucidating how these modules are coordinated and identifying the molecular drivers that cause microglia to transition to a dysfunctional state are crucial for understanding the pathology of ALS.

Immune checkpoint molecules, inhibitory receptors best known for their roles in T cell exhaustion and cancer immunotherapy [21, 22], have emerged as candidate regulators of microglial functional transitions. A recent study demonstrated that TIM-3 governs the homeostatic-to-DAM transition in microglia in an Alzheimer’s disease model [23], establishing that immune checkpoint molecules can regulate microglial activation states during neurodegeneration.

LAG-3 (lymphocyte activation gene-3, CD223) is an inhibitory receptor originally identified on T cells [21, 24, 25]. We recently demonstrated that LAG-3 is expressed on microglia and functions as an anti-inflammatory checkpoint, with its knockdown enhancing inflammatory responses [26]. However, whether LAG-3 actively regulates microglial functional states during neurodegeneration, and whether its contribution varies with disease stage, has not been addressed.

In the present study, we investigated the role of LAG-3 in microglial regulation during ALS pathogenesis using SOD1^G93A^ mice [27]. Our findings demonstrate that LAG-3 acts as a stage-dependent regulator that dissociates the inflammatory and phagocytic modules within the DAM program, providing a molecular basis for the stage-dependent functional transitions of microglia observed during ALS progression.

## Methods

### Mice

All experiments were reviewed and approved by the Keio University Animal Care and Use Committee, and care was taken to minimize suffering and limit the number of animals used. Transgenic mice carrying the human mutant SOD1^G93A^ gene (B6.Cg-Tg(SOD1*G93A)1Gur/J) and mice with targeted disruption of the LAG-3 gene (B6.129S6(Cg)-Lag3tm1Doi/J) were purchased from Jackson Laboratory.

### Experimental design

Heterozygous SOD1^G93A^ mice were crossed with LAG-3-heterozygous mice to generate SOD1^G93A^/LAG-3^+/+^, SOD1^G93A^/LAG-3^+/−^, and SOD1^G93A^/LAG-3^−/−^ offspring as littermates. Genotypes for *Lag3* and SOD1^G93A^ were determined by polymerase chain reaction (PCR) using genomic DNA extracted from tail biopsies.

To analyze mouse phenotype and survival, SOD1^G93A^/LAG-3^−/−^ and SOD1^G93A^/LAG-3^+/−^ mice were always compared with their SOD1^G93A^/LAG-3^+/+^ littermates. Non-transgenic littermates served as wild-type (WT) controls in tissue analysis experiments. Body weight of each mouse was monitored every 2–3 days. Disease onset was retrospectively determined as the time when mice reached peak body weight. Disease end-stage was defined as the time at which the animal could not right itself within 30 s after being placed on its side. Disease duration was defined as the interval between disease onset and end-stage. Sexes were balanced in each experimental group when analyzing life-span to avoid possible bias from sex-related intrinsic disease severity [28].

### Rotarod test

The Rotor Rod Test was conducted once a week from 12 weeks of age to endpoint with a device (MK-610A, Muromachi Kikai). The mice were able to stay on the rotating wheel at a constant speed of 15 rpm for up to 180 s. The test was conducted three times until the mice fell off the wheel, and the average value was recorded.

### Real-time quantitative PCR

Lumbar spinal cord (L2–L5), cervical lymph nodes, lumbar lymph nodes, and spleen were dissected from mice at each disease stage timepoint and immediately frozen in liquid nitrogen. Total RNA was extracted from each tissue using FastGene™ RNA Basic Kit (Nippon Genetics) according to the manufacturer’s instructions. Reverse transcription (1.5 µg of RNA) and quantitative PCR were performed with PrimeScript™ RT Master Mix (Takara Bio) and KAPA SYBR Fast qPCR Kit (Roche) using a Thermal Cycler Dice Real Time System II (TP950; Takara Bio): initial denaturation at 95 °C for 30 s was followed by 40 cycles of 95 °C for 5 s and 60 °C for 30 s, and a final dissociation stage entailing 95 °C for 15 s, 60 °C for 15 s, and 95 °C for 15 s. Primer sequences are listed in Supplementary Table 1. Relative gene expression normalized to mouse *Gapdh* was determined using the ΔΔCt method. All reactions were conducted in duplicate.

### Western blot

For Western blot analysis of LAG-3, Dectin-1, and β-actin, lumbar spinal cord tissue and cultured primary microglia were homogenized in lysis buffer (40 mM HEPES, pH 7.4, 150 mM NaCl, 10% glycerol, 1% Triton X-100, 0.5% sodium deoxycholate, 0.1% SDS) supplemented with 1 × protease inhibitor cocktail (Nacalai Tesque). Protein concentrations were determined by the BCA assay. Aliquots containing 30 µg of protein were subjected to SDS–polyacrylamide gel electrophoresis and transferred to polyvinylidene difluoride (PVDF) membranes (Immobilon-P; Merck Millipore). The membranes were blocked for 30 min at room temperature in Tris-buffered saline (TBS; 10 mM Tris–HCl, pH 7.5, and 150 mM NaCl) containing 0.05% Tween-20 (TBS-T) with 5% non-fat skim milk. The membranes were then incubated overnight at 4 °C with primary antibodies as follows: rabbit monoclonal anti-LAG-3 antibody (1:1,000, ab209238; Abcam), rabbit monoclonal anti-Dectin-1 antibody (1:1,000, 30260; CST), and mouse monoclonal anti-β-actin antibody (1:3,000, MAB1501; Merck). The membranes were incubated with appropriate horseradish peroxidase-conjugated secondary antibodies (Bio-Rad). Proteins were detected using ECL Prime Western Blotting Detection Reagent (Cytiva) and images were acquired using an ODYSSEY Fc Imaging System (LI-COR Biosciences).

### Immunohistochemistry

Mice were anesthetized with isoflurane and perfused transcardially with phosphate-buffered saline (PBS), followed by 4% paraformaldehyde (PFA) in 0.1 M phosphate buffer at pH 7.4 (PB). The spinal cord was dissected and postfixed in the same fixative overnight at 4 °C, then immersed in 20% sucrose in PB overnight at 4 °C. The tissue was then frozen in OCT compound (Sakura Finetek). The lumbar region of the spinal cord was serially sectioned at 40 µm using a cryostat. Sections were immunohistochemically processed as previously described [29]. For confocal scanning fluorescence microscopy, sections were incubated with primary antibodies, then with Alexa Fluor-conjugated secondary antibodies (1:200; Invitrogen), and mounted in Fluoromount-G (Southern Biotechnology).

The primary antibodies and dilutions used were as follows: rabbit polyclonal anti-Iba1 (1:1,000, 019–19741; FUJIFILM Wako), goat polyclonal anti-Iba1 (1:500, 011–27991; FUJIFILM Wako), rabbit monoclonal anti-LAG-3 antibody (1:1,000, ab209238; Abcam), mouse monoclonal anti-GFAP (1:3,000, G3893; Sigma-Aldrich), rat monoclonal anti-Dectin-1 (1:500, mabg-mdect-2; InvivoGen), goat polyclonal anti-Axl (1:500, AF854; R&D Systems), rat monoclonal anti-CD68 (1:1,000, MCA1957; Bio-Rad), goat polyclonal anti-ChAT (1:500, AB144P; Sigma-Aldrich) and rat monoclonal anti-LAMP1 (1:200, 121601; Biolegend). Sections were examined using an Olympus FV-3000 and FV-4000 confocal microscope system (Tokyo, Japan). Quantitative analyses of fluorescence images were performed using Fiji (ImageJ), and three spinal cord sections per animal were analyzed.

For the assessment of motor neuron number, cells with ChAT^+^ cytoplasm located in the ventral horn were deemed to be motor neurons. For microglial morphology and CD68 immunoreactivity analysis, LAG-3-high and LAG-3-low Iba1^+^ cells were classified based on LAG-3 fluorescence intensity. Individual Iba1^+^ cells were outlined in Fiji, and morphological parameters (soma area, circularity, solidity) were calculated using the Analyze Particles function. CD68 immunoreactivity in LAG-3-high and LAG-3-low Iba1^+^ cells was quantified as mean fluorescence intensity within each cell outline, measured in Fiji.

### Flow cytometry and FACS sorting

Mice were anesthetized with isoflurane and perfused transcardially with PBS. The lumbar spinal cord was dissected and collected in DMEM high-glucose medium supplemented with 10% fetal bovine serum (FBS). Tissues were mechanically dissociated by trituration on ice and passed through a 100 µm cell strainer. To isolate immune cells, the cell suspension was resuspended in 37% Percoll PLUS (Cytiva) and centrifuged at 780 × g for 20 min. After centrifugation, the myelin debris layer was removed, and the cell pellet containing immune cells, including microglia, was collected. Isolated cells were washed with PBS containing 2% FBS and 0.1% sodium azide, and Fc receptors were blocked with an anti-CD16/CD32 antibody prior to cell surface labeling. Cell surface staining was performed for 30 min at 4 °C using the following antibodies: anti-CD45-PE, anti-CD11b-FITC, anti-CD25-BV421, anti-NK1.1-PE-Cy7 (Biolegend), anti-CD45-BUV805, anti-CD11b-BV605, anti-CD3-PerCP, anti-CD4-PE, anti-CD8-FITC (BD Biosciences), and anti-LAG-3-Alexa Fluor 647 conjugate (CST). Microglia were identified as CD11b^+^/CD45^int^. Infiltrating lymphoid populations were identified as: CD8 + T cells (CD45^hi^/CD3^+^/CD8^+^), CD4 + T cells (CD45^hi^/CD3^+^/CD4^+^/CD25^−^), regulatory T cells (CD45^hi^/CD3^+^/CD4^+^/CD25^+^), and NK cells (CD45^hi^/CD3^−^/NK1.1^+^). Dead cells were identified by staining with propidium iodide. Flow cytometry analysis and cell sorting were performed using a BD FACSAria III and BD FACSymphony A5 (BD Biosciences), and data were analyzed using FlowJo software (Tree Star). For RNA-seq experiments, microglia were further sorted into LAG-3-high and LAG-3-low populations based on LAG-3 fluorescence intensity, with gating thresholds defined according to the fluorescence minus one (FMO) control and the LAG-3 intensity distribution.

### RNA-sequencing

#### Microglia RNA-seq

For microglial transcriptomic analyses, CD11b^+^/CD45^int^ microglia were sorted from the lumbar spinal cord using a BD FACSAria III cell sorter. For the characterization of LAG-3-expressing microglia, microglia from P140 SOD1^G93A^ mice were further separated into LAG-3-high and LAG-3-low populations, with WT microglia serving as controls. For the stage-dependent analysis, CD11b^+^/CD45^int^ microglia were sorted from SOD1^G93A^/LAG-3^+/+^ and SOD1^G93A^/LAG-3^−/−^ mice at both P120 and P140. RNA-sequencing libraries were prepared using the SMART-seq mRNA HT LP Kit (Takara Bio) according to the manufacturer’s instructions. The libraries were sequenced on an Illumina NovaSeq X Plus platform with paired-end 150 bp reads.

#### Spinal cord bulk RNA-seq

For bulk RNA-seq analysis of the lumbar spinal cord, total RNA was extracted from P140 SOD1^G93A^/LAG-3^+/+^ and SOD1^G93A^/LAG-3^−/−^ lumbar spinal cord tissue as described in the qPCR section. Sequencing libraries were prepared using the NEBNext Ultra II Directional RNA Library Prep Kit (New England Biolabs) according to the manufacturer’s instructions. The libraries were sequenced on an Illumina NovaSeq X Plus platform with paired-end 150 bp reads.

### Bioinformatics analysis

Raw sequencing reads were processed with Trim Galore (version 0.6.7) for quality control and adapter trimming. Transcript abundance was quantified using Salmon (version 1.9.0) against the mouse reference transcriptome (GRCm39). Count matrices were generated using tximport in R (v4.2.1). Principal component analysis (PCA) was performed using the prcomp function on log-transformed, variance-stabilized count data.

Differential gene expression analysis was performed using DESeq2 (v1.48.2). For the LAG-3-high versus LAG-3-low comparison and the P140 spinal cord comparison, genes with an adjusted *p*-value < 0.1 and |log2 fold change|> 1 were considered differentially expressed. For the stage-dependent microglial analysis, a two-way ANOVA-like model was fitted with DESeq2, using genotype (LAG-3^+/+^ vs LAG-3^−/−^) and disease stage (P120 vs P140) as factors, with their interaction term included. This design enabled identification of genes affected by genotype, disease stage, and the genotype-by-stage interaction, the last of which specifically isolates stage-dependent effects of LAG-3 deletion. Genes with significant interaction terms were further classified by the direction of the interaction coefficient to distinguish genes upregulated or downregulated by LAG-3 deletion specifically at the late disease stage.

Gene Ontology (GO) enrichment analysis of biological processes and Kyoto Encyclopedia of Genes and Genomes (KEGG) pathway enrichment analysis were performed using clusterProfiler (v4.16.0) in R. Volcano plots and heatmaps were generated using the ggplot2 (v4.0.1) and ComplexHeatmap (v2.24.1) packages, respectively. For heatmap visualization, gene expression values were z-score normalized across samples.

### Primary microglia isolation from adult spinal cord

Primary microglia were isolated from the lumbar spinal cord of SOD1^G93A^/LAG-3^+/+^ and SOD1^G93A^/LAG-3^−/−^ mice at the early symptomatic (P120) and late symptomatic (P140) stages. The isolation method followed the modified protocol for the brain of adult mice [30], adapted for spinal cord samples. Mice were anesthetized with isoflurane and perfused transcardially with PBS. The lumbar spinal cord was rapidly dissected and minced in an enzyme digestion mix (2 mg/mL collagenase type A, 70 U/mL DNase I, 10 mM HEPES and 5% FBS in HBSS). The tissue was incubated at 37 °C for 15 min with gentle agitation every 5 min, then sequentially dissociated to a single-cell suspension using two flame-polished Pasteur pipettes of decreasing bore size. The cell suspension was filtered through a sterile 70 µm nylon cell strainer and centrifuged at 300 × g for 10 min at 4 °C. The cell pellet was resuspended in 37% Percoll PLUS and centrifuged at 780 × g for 20 min. After centrifugation, the myelin debris layer was removed and the cell pellet was washed with PBS at 300 × g for 10 min. Red blood cells were lysed by incubation in Hemolysis Buffer for 2 min at room temperature, followed by washing in PBS. Cells were seeded in T25 flasks in complete medium (DMEM/F-12 supplemented with 10% FBS, 1% GlutaMAX and 1% penicillin/streptomycin). After 3 h, non-adherent cells were removed by medium exchange. The following day, recombinant GM-CSF and M-CSF were added at a final concentration of 100 ng/mL each to support microglial proliferation. On day 3 post-seeding, the medium was replaced with fresh medium supplemented with the same concentrations of GM-CSF and M-CSF. The cells were maintained for a total of 7 days before being used in functional assays.

### Phagocytosis assay

The phagocytic capacity of primary microglia was assessed using pHrodo™ Deep Red E. coli BioParticles™ Conjugate (Thermo Fisher Scientific). Primary microglia isolated from SOD1^G93A^/LAG-3^+/+^ and SOD1^G93A^/LAG-3^−/−^ mice at the early symptomatic and late symptomatic stages were seeded in 96-well plates (2 × 10^4^ cells/well) and allowed to adhere for 24 h. Cells were then incubated with fluorescent bioparticles at 500 µg/mL, and the plates were transferred to a NIVO plate reader (Revvity) maintained at 37 °C. Fluorescence intensity was recorded every 30 min for 4 h. At each time point, background fluorescence from beads-only control wells was subtracted from the experimental values to calculate net phagocytic uptake. The area under the curve (AUC) was calculated over the 0–4 h period to quantify cumulative phagocytic activity.

### Generation of mouse Lag3 expression plasmid and LAG-3 rescue transfection experiment

A mouse Lag3 expression plasmid (pCMV-mLag3) was constructed by replacing the DsRed-Monomer coding sequence of the pDsRed-Monomer-C1 vector (Takara Bio) with the full-length mouse Lag3 open reading frame using standard molecular cloning techniques. The construct expresses mLag3 under control of the CMV promoter. An empty vector lacking both DsRed-Monomer and Lag3 sequences was used as the mock control. All constructs were verified by Sanger sequencing.

Primary microglia isolated from P140 SOD1^G93A^/LAG-3^−/−^ mice were cultured for 7 days as described above. On day 7, cells were transfected with either pCMV-mLag3 or empty vector control using Lipofectamine 3000 according to the manufacturer's instructions. 0.25 µg of plasmid DNA was delivered per well in 96-well format. At 72 h post-transfection, transfection efficiency and LAG-3 expression were confirmed by Western blot analysis. Phagocytosis assay was performed at 72 h post-transfection as described above.

### Immunocytochemistry

For immunocytochemical analysis of phagocytic uptake, primary microglia from SOD1^G93A^/LAG-3^+/+^ and SOD1^G93A^/LAG-3^−/−^ mice were seeded in 96-well plates (2 × 10^4^ cells/well) and allowed to adhere for 24 h. Cells were incubated with pHrodo™ Deep Red E. coli BioParticles™ Conjugate (500 µg/mL) for 2 h, then fixed and immunostained as previously described [26]. For confocal fluorescence microscopy, cells were incubated with rabbit polyclonal anti-Iba1 antibody (1:1,000, 019–19741; FUJIFILM Wako) as the primary antibody, followed by an Alexa Fluor-conjugated secondary antibody (1:400; Invitrogen) and DAPI (1:3,000; Molecular Probe). Cells were mounted in Fluoromount-G (Southern Biotechnology) and examined using an Olympus FV-3000 confocal microscope system.

### Statistical analysis

All statistical analyses were performed using GraphPad Prism software (v10.5.0). Data are presented as mean ± standard error of the mean (SEM). For comparisons among three or more groups, one-way analysis of variance (ANOVA) was used followed by Tukey’s multiple comparisons test for all pairwise comparisons, or Dunnett’s multiple comparisons test when comparisons were made against a single control group. For experiments with two independent variables (e.g., genotype and disease stage), two-way ANOVA was performed followed by Bonferroni's multiple comparisons test for all pairwise comparisons. Survival curves were analyzed using the Kaplan–Meier method and compared by the log-rank (Mantel–Cox) test. Comparisons between two groups were performed using an unpaired Student’s t-test. Statistical significance was defined as *p* < 0.05. The specific statistical tests and sample sizes for each experiment are detailed in the corresponding figure legends.

## Results

### LAG-3 is upregulated in spinal microglia during disease progression of SOD1^G93A^ mice

**Fig. 1:**
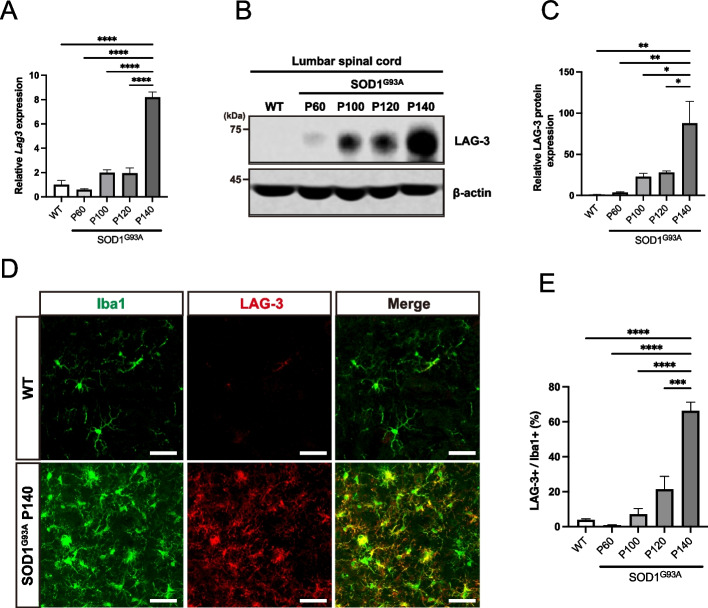
LAG-3 is upregulated in spinal microglia during disease progression of SOD1^G93A^ mice. **A** Quantitative PCR analysis of LAG-3 mRNA in lumbar spinal cord showing progressive increase during disease progression with dramatic elevation at P140. *n* = 3 per group. Mean ± SEM. ****p < 0.0001 by one-way ANOVA with Tukey's multiple comparisons test. **B** Representative Western blots showing LAG-3 and β-actin in lumbar spinal cord lysates. **C** Densitometric quantification of LAG-3 showing progressive accumulation with prominent upregulation at P140. *n* = 3 per group. Mean ± SEM. *p < 0.05, **p < 0.01 by one-way ANOVA with Tukey's multiple comparisons test. **D** Representative immunofluorescence images of LAG-3 and Iba1 in lumbar spinal cord ventral horn from WT and SOD1^G93A^ P140. LAG-3 is predominantly localized to Iba1^+^ microglia/macrophages. Scale bars: 40 μm. **E** Quantification of LAG-3^+^/Iba1^+^ double-positive area showing progressive increase during disease progression with marked elevation at P140. n = 3 mice per group. Mean ± SEM. ***p < 0.001, ****p < 0.0001 by one-way ANOVA with Tukey's multiple comparisons test

We first characterized the spatiotemporal expression of LAG-3 in the lumbar spinal cord of SOD1^G93A^ mice. LAG-3 mRNA levels increased progressively as the disease advanced, with marked upregulation at the late symptomatic stage (P140) (Fig. 1A). Consistent with this transcriptional increase, LAG-3 protein levels were also elevated, as shown by Western blot analysis (Fig. 1B-C).

To determine whether this LAG-3 upregulation is restricted to the central nervous system, we examined LAG-3 mRNA levels in peripheral lymphoid tissues throughout disease progression. In contrast to the spinal cord, *Lag3* expression in cervical lymph nodes, lumbar lymph nodes, and spleen remained unchanged from presymptomatic through late symptomatic stage (Sup. Fig. 1A-C).

We next sought to identify which cell types within the spinal cord express LAG-3 during the disease. Flow cytometric analysis of immune cell populations in P140 SOD1^G93A^ spinal cord revealed that microglia had significantly higher LAG-3 expression compared to other immune cells examined, including infiltrating T cell subsets (CD8^+^, CD25^−^/CD4^+^, CD25^+^/CD4^+^ Treg) and NK cells (Sup. Fig. 1D, E). This identifies microglia as the dominant LAG-3-expressing immune cell type within the diseased spinal cord. Consistent with this finding, immunohistochemical analysis demonstrated that LAG-3 immunoreactivity in spinal cord sections colocalized with Iba1 (Fig. 1D), with progressively increasing LAG-3^+^ microglia/macrophages during disease progression (Fig. 1E). Within the spinal cord, LAG-3-expressing Iba1^+^ cells were predominantly distributed in the ventral horn and intermediate zone, where motor neuron degeneration occurs, with minimal expression in the dorsal horn (Sup. Fig. 1F).

### LAG-3-high microglia exhibit disease-associated microglia signature

**Fig. 2:**
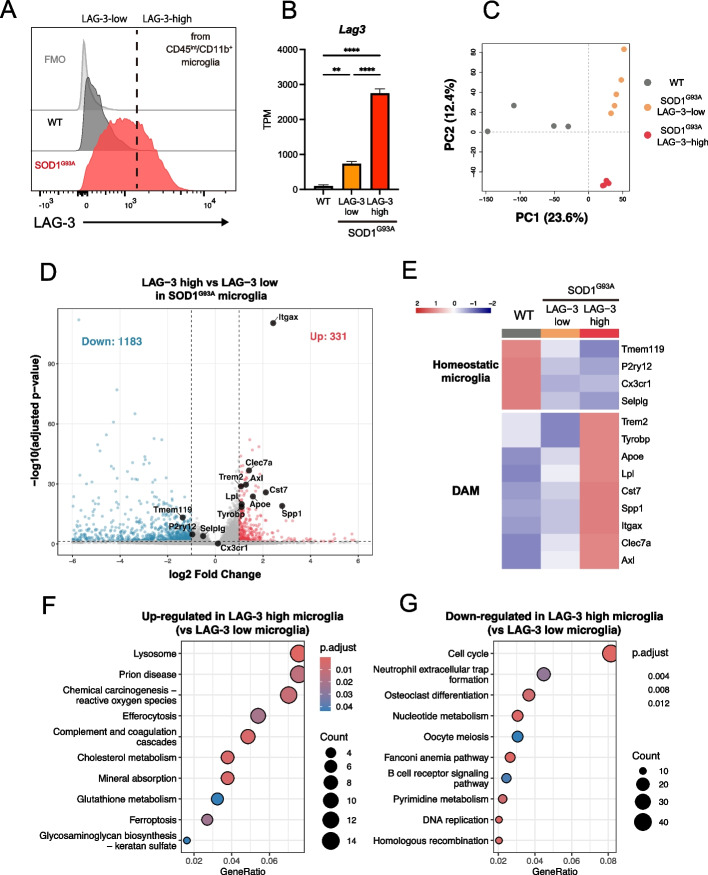
LAG-3-high microglia exhibit disease-associated microglia signature. **A** Representative flow cytometry histogram showing LAG-3 expression on CD11b^+^/CD45^int^ spinal cord microglia from WT and SOD1^G93A^ P140 mice. FMO (fluorescence minus one) control is shown in light gray. Dashed lines indicate gating thresholds defining LAG-3-low and LAG-3-high populations. **B** Quantitative validation of *Lag3* transcript levels (TPM, Transcripts Per Million) in sorted microglial subpopulations. Mean ± SEM. **p < 0.01, ****p < 0.0001 by one-way ANOVA followed by Tukey's post-hoc test. **C** Principal component analysis (PCA) of transcriptomes from WT microglia (*n* = 4), SOD1^G93A^ LAG-3-low (*n* = 5), and SOD1^G93A^ LAG-3-high (*n* = 5) microglia. **D** Volcano plot showing differentially expressed genes (DEGs) between LAG-3-high and LAG-3-low microglia from SOD1^G93A^ mice. DAM and homeostatic microglial markers are labeled. **E** Heatmap displaying z-score normalized expression of homeostatic and DAM marker genes across WT, LAG-3-low, and LAG-3-high microglia. **F**, **G** KEGG pathway enrichment analysis of genes upregulated (**F**) or downregulated (**G**) in LAG-3-high microglia

To examine the transcriptional identity of LAG-3-expressing microglia, we isolated microglia from the spinal cord of P140 SOD1^G93A^ mice by FACS. CD11b^+^/CD45^int^ microglia (Sup. Fig. 2A) displayed broad LAG-3 immunoreactivity, with overall expression markedly higher in SOD1^G93A^ than in WT microglia (Fig. 2A). We defined gating thresholds to separate LAG-3-high and LAG-3-low subpopulations (Fig. 2A) and isolated each fraction alongside WT microglia for RNA-seq. *Lag3* transcript levels progressively increased from WT through LAG-3-low to LAG-3-high microglia (Fig. 2B). In addition, *Havcr2* (encoding TIM-3) was higher in LAG-3-high than in LAG-3-low microglia (Sup. Fig. 2B).

Principal component analysis (PCA) revealed clear transcriptional separation among WT microglia, SOD1^G93A^ LAG-3-low microglia, and SOD1^G93A^ LAG-3-high microglia, with PC1 (23.6%) primarily separating disease from non-disease states and PC2 (12.4%) distinguishing LAG-3 expression levels (Fig. 2C). Differential gene expression analysis identified 1,183 downregulated and 331 upregulated genes in LAG-3-high compared with LAG-3-low microglia (Fig. 2D). LAG-3-high microglia exhibited elevated expression of established disease-associated microglia (DAM) markers, including *Trem2, Tyrobp, Apoe, Lpl, Cst7, Spp1, Itgax, Clec7a,* and *Axl,* while showing reduced expression of homeostatic markers such as *Tmem119, P2ry12, Cx3cr1,* and *Selplg* (Fig. 2D). Heatmap analysis further revealed a progressive shift in microglial phenotype from homeostatic (WT) to intermediate (LAG-3-low) to fully activated DAM state (LAG-3-high) (Fig. 2E).

We then validated the DAM identity of LAG-3-high microglia at the protein and morphological levels *in vivo*. Triple immunostaining of LAG-3, Iba1, and CD68 demonstrated that LAG-3-high Iba1^+^ cells exhibited significantly higher CD68 immunoreactivity than LAG-3-low Iba1^+^ cells (Sup. Fig. 2C, D). Furthermore, LAG-3-high Iba1^+^ cells displayed marked morphological alterations, including an enlarged soma, increased circularity, and increased solidity, which are characteristic features of an activated, amoeboid-like morphology (Sup. Fig. 2E–G). These histological characteristics align with the transcriptional DAM signature and demonstrate that LAG-3-high microglia recapitulate features of DAM.

KEGG pathway enrichment analysis comparing LAG-3-high to LAG-3-low microglia revealed that upregulated genes were associated with lysosome, efferocytosis, cholesterol metabolism, complement and coagulation cascades, and oxidative stress responses (Fig. 2F), while downregulated genes were enriched in cell cycle-related pathways including DNA replication, homologous recombination, and nucleotide metabolism (Fig. 2G). Comparison with WT microglia confirmed prominent enrichment of phagocytic and lysosomal pathways (Lysosome biogenesis, Phagosome, Efferocytosis, Autophagy, Mitophagy) and lipid metabolism (Sup. Fig. 2H–J), along with downregulation of cell proliferation and leukocyte migration processes.

Collectively, these results demonstrate that LAG-3-high microglia exhibit a reactive, disease-associated microglial signature, characterized by upregulation of canonical DAM markers and a morphologically activated state.

### LAG-3 deletion induces a biphasic phenotype and stage-dependent pathology in SOD1^G93A^ mice

**Fig. 3:**
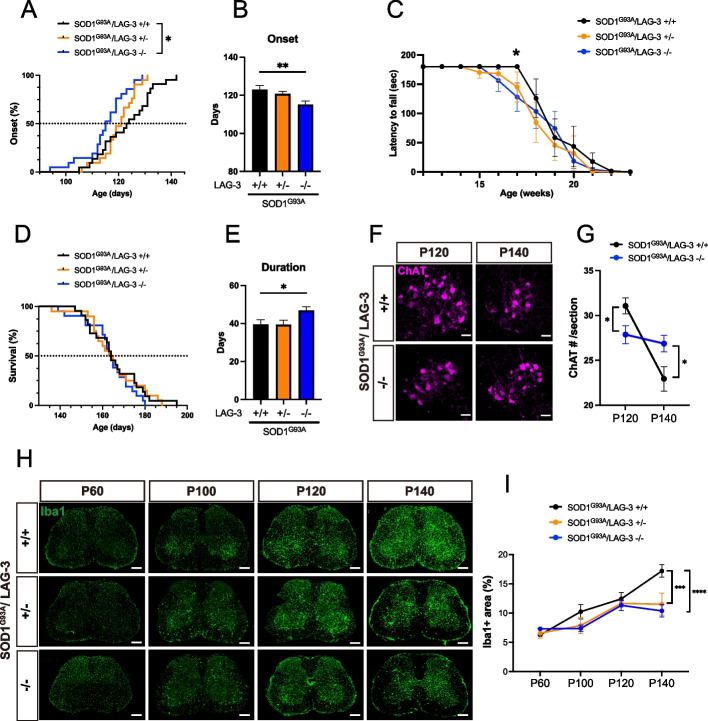
LAG-3 deletion induces a biphasic phenotype and stage-dependent pathology in SOD1^G93A^ mice. **A** Kaplan–Meier curve showing disease onset. SOD1^G93A^/LAG-3^+/+^: *n* = 22, SOD1^G93A^/LAG-3^+/-^: *n* = 20, SOD1^G93A^/LAG-3^−/−^:*n* = 21. *p < 0.05 by log-rank test. **B** Mean disease onset time. Mean ± SEM. **p < 0.01 by one-way ANOVA with Dunnett’s multiple comparisons test (vs SOD1^G93A^/LAG-3^+/+^). **C** Evaluation of motor performance by rotarod test. SOD1^G93A^/LAG-3^+/+^: *n* = 5, SOD1^G93A^/LAG-3^+/-^: *n* = 6, SOD1^G93A^/LAG-3^−/−^:*n* = 7. *p < 0.05 by two-way ANOVA with Bonferroni's multiple comparisons test (SOD1^G93A^/LAG-3^+/+^ vs SOD1^G93A^/LAG-3^−/−^). **D** Kaplan–Meier survival curves showing overall survival across genotypes. SOD1^G93A^/LAG-3^+/+^: *n* = 22, SOD1^G93A^/LAG-3^+/-^: *n* = 20, SOD1^G93A^/LAG-3^−/−^:*n* = 21. **E** Disease duration (survival age minus onset age) is significantly extended in LAG-3^−/−^ mice. Mean ± SEM. *p < 0.05 by one-way ANOVA with Dunnett’s multiple comparisons test (vs SOD1^G93A^/LAG-3^+/+^). **F** Representative immunofluorescence images of ChAT in lumbar spinal cord across genotypes and disease stages. Scale bars: 40 μm. **G** Quantification of the number of ChAT^+^ motor neurons in lumbar spinal cord across genotypes and disease stages. n = 5 mice per group. Mean ± SEM. *p < 0.05 by Student's t-test. **H** Representative immunofluorescence images of Iba1 in lumbar spinal cord across genotypes and disease stages. Scale bars: 200 μm. **I** Quantification of Iba1^+^ area in lumbar spinal cord across genotypes and disease stages. *n* = 3 mice per genotype at P60 and P100; at P120 and P140, *n* = 6 mice for SOD1^G93A^/LAG-3^+/+^ and SOD1^G93A^/LAG-3^−/−^, and *n* = 5 mice for SOD1^G93A^/LAG-3^+/-^. Mean ± SEM. ***p < 0.001, ****p < 0.0001 by two-way ANOVA with Bonferroni's multiple comparisons test

To investigate the functional role of LAG-3 in ALS pathogenesis, we generated SOD1^G93A^/LAG-3^−/−^ mice. Survival analysis revealed a paradoxical biphasic phenotype: LAG-3 deficiency accelerated disease onset (Fig. 3A, B), accompanied by earlier dropout from rotarod testing (Fig. 3C), yet overall survival time remained unchanged across genotypes (Fig. 3D). Disease duration, defined as the interval between onset and death, was significantly extended in SOD1^G93A^/LAG-3^−/−^ mice compared with SOD1^G93A^/LAG-3^+/+^ controls (Fig. 3E). Thus, LAG-3 deletion produced opposing effects on the temporal course of SOD1^G93A^ mice: acceleration of early-phase symptoms followed by prolongation of the subsequent disease course.

To examine motor neuron pathology underlying this biphasic disease phenotype, we quantified ChAT^+^ motor neurons in the lumbar ventral horn at early (P120) and late (P140) symptomatic stages (Fig. 3F). At P120, motor neuron counts were reduced in SOD1^G93A^/LAG-3^−/−^ mice compared to SOD1^G93A^/LAG-3^+/+^ controls, whereas at P140, this relationship was reversed, with SOD1^G93A^/LAG-3^−/−^ mice retaining more motor neurons than SOD1^G93A^/LAG-3^+/+^ mice (Fig. 3G). This biphasic profile reflected progressive motor neuron loss in SOD1^G93A^/LAG-3^+/+^ mice, whereas SOD1^G93A^/LAG-3^−/−^ mice showed preserved counts across stages.

We next investigated the glial response. Iba1 immunohistochemistry across disease stages (P60–P140) revealed progressive accumulation of Iba1^+^ microglia/macrophages in SOD1^G93A^/LAG-3^+/+^ spinal cord (Fig. 3H, I). No genotype-dependent differences in Iba1^+^ area were detected at P60, P100, or P120. At P140, however, Iba1^+^ area was markedly reduced in SOD1^G93A^/LAG-3^−/−^ mice compared with SOD1^G93A^/LAG-3^+/+^ controls, indicating that LAG-3 deficiency suppresses microgliosis selectively at the late disease stage. Consistent with this late-stage suppression, Western blot analysis of the P140 spinal cord revealed that Dectin-1 expression, which was undetectable in WT but markedly elevated in SOD1^G93A^/LAG-3^+/+^ tissue, was significantly reduced in the SOD1^G93A^/LAG-3^−/−^ spinal cord (Sup. Fig. 3A, B). In contrast, GFAP immunostaining showed no significant differences in astrocyte activation across genotypes at any disease stage (Sup. Fig. 3C, D).

These results demonstrate that LAG-3 deletion produces a stage-dependent biphasic phenotype in SOD1^G93A^ mice, with concordant patterns observed in both behavioral and histopathological analyses.

### LAG-3 deficiency exerts stage-dependent effects on microglial gene expression

To characterize the transcriptional changes underlying stage-dependent phenotypes in LAG-3-deficient mice, we performed RNA-seq analysis on microglia from SOD1^G93A^/LAG-3^+/+^ and SOD1^G93A^/LAG-3^−/−^ mice at early (P120) and late (P140) disease stages. This 2 × 2 factorial design enabled us to dissect genotype effects, temporal effects, and their interaction.

**Fig. 4:**
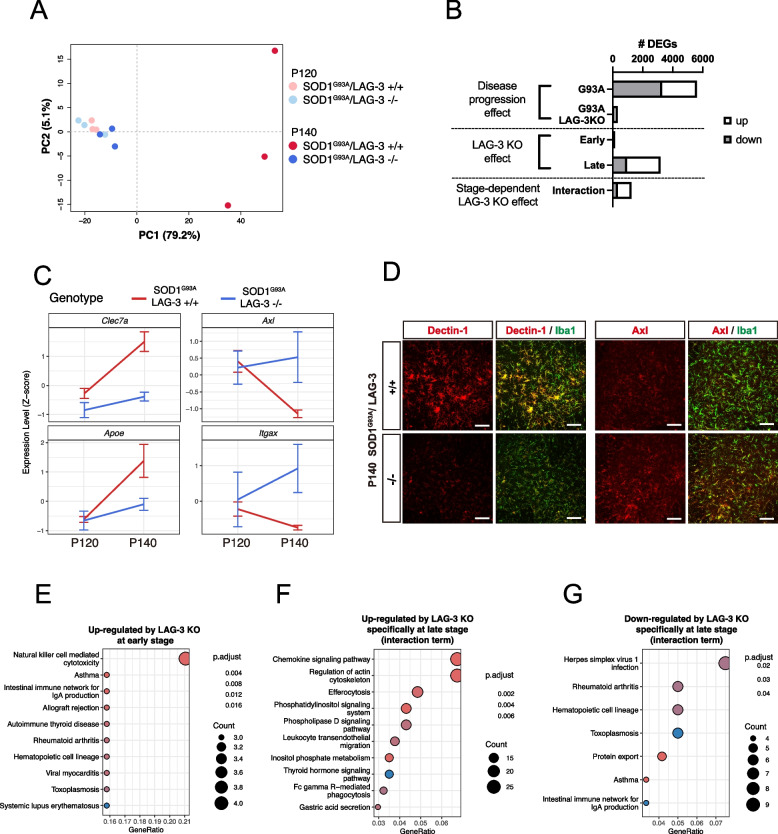
LAG-3 deficiency exerts stage-dependent effects on microglial gene expression. **A** Principal component analysis (PCA) of microglial transcriptomes from P120 and P140 in SOD1^G93A^/LAG-3^+/+^ and SOD1^G93A^/LAG-3^−/−^. *n* = 3 per group. **B** Summary of differentially expressed genes (DEGs) from two-way ANOVA analysis. Bars show number of upregulated and downregulated genes for disease progression effect (P140 vs P120 in each genotype), LAG-3 KO effect at early stage (P120), LAG-3 KO effect at late stage (P140), and genotype-by-stage interaction (stage-dependent LAG-3 KO effect). **C** Expression trajectories of representative DAM markers showing stage-dependent responses to LAG-3 deletion (z-score normalized). *n* = 3 per group. Mean ± SEM. **D** Representative immunofluorescence images showing Dectin-1 (*Clec7a*) and Axl expression in Iba1^+^ microglia/macrophages in lumbar spinal cord from P140 SOD1^G93A^/LAG-3^+/+^ and SOD1^G93A^/LAG-3^−/−^. Scale bars: 40 μm. **E** KEGG pathway enrichment analysis of genes upregulated in P120 SOD1^G93A^/LAG-3^−/−^ microglia compared to P120 SOD1^G93A^/LAG-3^+/+^. **F**, **G** KEGG pathway enrichment analysis of genes upregulated (**F**) or downregulated (**G**) by LAG-3 deletion specifically at late stage (interaction term)

Principal component analysis revealed that P140 SOD1^G93A^ microglia were distinctly separated from the other three groups along PC1 (79.2%), while P120 and P140 LAG-3^−/−^ samples clustered more closely together (Fig. 4A). Differential expression analysis identified substantial transcriptional changes associated with disease progression in SOD1^G93A^ microglia (P140 vs P120), modest changes due to LAG-3 deletion at the early stage (P120), pronounced effects of LAG-3 deletion at the late stage (P140), and importantly, interaction effects indicating a stage-dependent LAG-3 KO effect (Fig. 4B).

Examination of representative DAM markers revealed that LAG-3 deletion suppressed the disease-associated upregulation of most DAM markers, including *Clec7a* and *Apoe*, during progression from P120 to P140. In contrast, phagocytosis-associated markers *Axl* and *Itgax* declined in SOD1^G93A^ microglia but were maintained or elevated in SOD1^G93A^/LAG-3^−/−^ microglia at P140 (Fig. 4C, Sup. Fig. 4A). Immunohistochemical analysis of lumbar spinal cord sections confirmed reduced Dectin-1(*Clec7a*) but increased Axl expression in P140 SOD1^G93A^/LAG-3^−/−^ Iba1^+^ cells (Fig. 4D), validating the transcriptional findings at the tissue level.

At the early disease stage (P120), LAG-3 deletion induced upregulation of inflammatory pathways including natural killer cell-mediated cytotoxicity and asthma-related immune responses, with no significantly downregulated pathways detected (Fig. 4E). Two-way ANOVA identified genes with significant genotype-by-stage interaction, isolating transcriptional changes specific to the late stage. Genes with positive interaction terms (upregulated by LAG-3 deletion specifically at P140) were enriched in pathways related to phagocytosis, efferocytosis, and chemokine signaling (Fig. 4F). Genes with negative interaction terms (downregulated by LAG-3 deletion specifically at P140) showed enrichment in inflammatory pathways, including rheumatoid arthritis and hematopoietic cell lineage (Fig. 4G). Importantly, the pathways identified in the interaction analysis (Fig. 4F, G) closely corresponded to those in the direct comparison between P140 SOD1^G93A^/LAG-3^−/−^ and SOD1^G93A^ microglia (Sup. Fig. 4B, C), confirming that the stage-dependent effects identified by the interaction term reflect late-stage-specific changes.

Together, these findings demonstrate that LAG-3 deletion enhanced inflammatory microglial responses at the early disease stage, whereas at the late stage it suppressed inflammatory gene expression while maintaining or upregulating phagocytic effector genes.

### LAG-3 deletion enhances microglial phagocytosis at the late disease stage

**Fig. 5:**
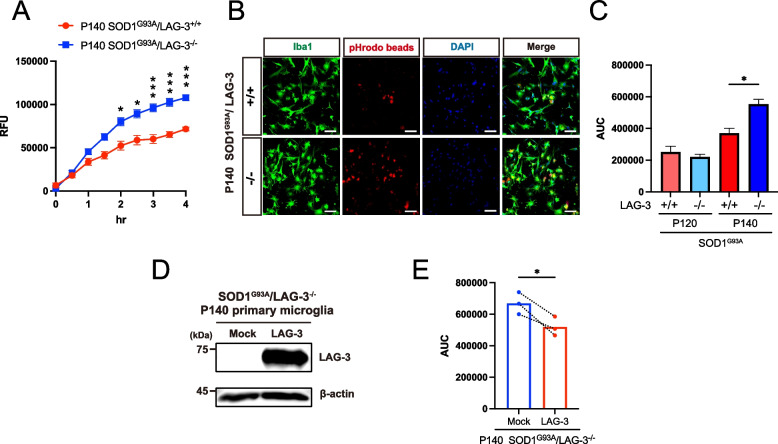
LAG-3 deletion enhances microglial phagocytosis at the late disease stage. **A**–**C** Functional validation of microglial phagocytic capacity using primary microglia isolated from the spinal cord. **A** Time course of phagocytic uptake assessed by pHrodo deep red beads assay in P140 SOD1^G93A^/LAG-3^+/+^ (*n* = 3) and SOD1^G93A^/LAG-3^−/−^ (*n* = 4) microglia. Mean ± SEM. *p < 0.05, ***p < 0.001 by two-way ANOVA with Bonferroni's multiple comparisons test. **B** Immunocytochemical analysis showing Iba1, internalized pHrodo beads, and DAPI in primary microglia after pHrodo bead treatment. Scale bars: 40 μm. **C** Quantification of the area under the curve (AUC) of pHrodo fluorescence intensity from 0 to 4 h in SOD1^G93A^/LAG-3^+/+^ and SOD1^G93A^/LAG-3^−/−^ microglia at P120 and P140 (*n* = 3 for P120 groups and P140 SOD1^G93A^/LAG-3^+/+^; *n* = 4 for P140 SOD1^G93A^/LAG-3^−/−^). Mean ± SEM. *p < 0.05 by two-way ANOVA with Bonferroni's multiple comparisons test. **D**–**E** Genetic rescue of phagocytic capacity by re-introducing LAG-3 into P140 SOD1^G93A^/LAG-3^−/−^ primary microglia. **D** Western blot analysis confirming the restoration of LAG-3 protein expression in SOD1^G93A^/LAG-3^−/−^ microglia transiently transfected with either a mock vector or a LAG-3-expressing plasmid. **E** Quantification of the area under the curve (AUC) of pHrodo fluorescence intensity from 0 to 4 h. Mean ± SEM. *n* = 3 per group. *p < 0.05 by paired t-test

To examine whether the transcriptional preservation of phagocytic effector genes in LAG-3-deficient microglia at the late disease stage (Fig. 4) translates into altered phagocytic function, we assessed the phagocytic capacity of primary microglia isolated from SOD1^G93A^/LAG-3^+/+^ and SOD1^G93A^/LAG-3^−/−^ mice using a pHrodo bead uptake assay. At the late symptomatic stage (P140), SOD1^G93A^/LAG-3^−/−^ microglia exhibited significantly higher pHrodo fluorescence intensity than SOD1^G93A^/LAG-3^+/+^ microglia throughout the assay period (Fig. 5A). Immunocytochemical analysis confirmed increased internalization of pHrodo beads within Iba1^+^ microglia from SOD1^G93A^/LAG-3^−/−^ mice (Fig. 5B). To test whether this enhancement was specific to the late stage, we compared the cumulative uptake (area under the curve, AUC) at both the early symptomatic (P120) and late symptomatic (P140) stages. At P140, AUC analysis confirmed increased phagocytic uptake in SOD1^G93A^/LAG-3^−/−^ microglia, whereas at P120, both genotypes exhibited comparable uptake with no significant difference (Fig. 5C).

To determine whether LAG-3 directly restrains phagocytic activity in a cell-autonomous manner, we performed rescue experiments by re-introducing LAG-3 into SOD1^G93A^/LAG-3^−/−^ microglia by transient transfection. Restoration of LAG-3 expression in transfected cells was confirmed by Western blot analysis (Fig. 5D). Compared to mock-transfected cells from the same preparation, LAG-3-rescued microglia exhibited reduced pHrodo uptake (Fig. 5E). This result demonstrates that LAG-3 functions cell-autonomously within microglia to restrain phagocytic uptake, and that loss of LAG-3 directly accounts for the enhanced phagocytic capacity of P140 SOD1^G93A^/LAG-3^−/−^ microglia.

### Improved lysosomal activity and spinal cord environment in SOD1^G93A^/LAG-3^−/−^ mice

**Fig. 6:**
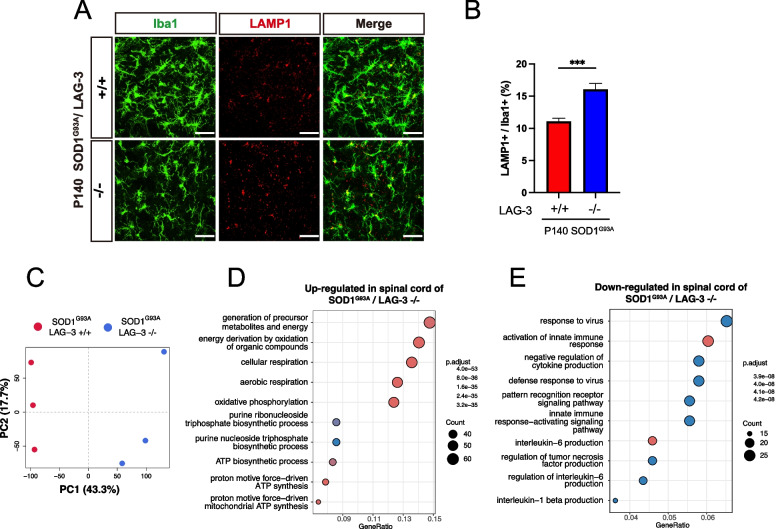
Improved lysosomal activity and spinal cord environment in SOD1^G93A^/LAG-3^−/−^ mice. **A** Representative immunofluorescence images showing LAMP1 expression in Iba1^+^ microglia/macrophages in lumbar spinal cord from P140 SOD1^G93A^/LAG-3^+/+^ and SOD1^G93A^/LAG-3^−/−^. Scale bars: 40 μm. **B** Quantification of LAMP1^+^/Iba1^+^ double-positive area in P140 SOD1^G93A^/LAG-3^+/+^ and SOD1^G93A^/LAG-3^−/−^. *n* = 6 mice per group. Mean ± SEM. ***p < 0.001, by Student's t-test. **C**–**E** Bulk RNA-seq analysis of lumbar spinal cord tissue from P140 SOD1^G93A^/LAG-3^+/+^and SOD1^G93A^/LAG-3^−/−^ mice (*n* = 3 each). **C** Principal component analysis (PCA) showing transcriptional separation between genotypes. **D**, **E** Gene Ontology (GO) enrichment analysis of biological processes for genes upregulated (**D**) or downregulated (**E**) in SOD1^G93A^/LAG-3^−/−^ spinal cord

To examine the in vivo consequences of LAG-3 deletion, we assessed microglial lysosomal activity in P140 spinal cord. The LAMP1^+^ area within Iba1^+^ microglia/macrophages was greater in SOD1^G93A^/LAG-3^−/−^ mice compared to SOD1^G93A^/LAG-3^+/+^ mice (Fig. 6A, B), indicating that LAG-3 deficiency enhances microglial lysosomal activity *in vivo*, consistent with the increased phagocytic capacity observed in vitro.

To assess the broader effects of LAG-3 deletion on the spinal cord environment, we performed bulk RNA-sequencing of lumbar spinal cord tissue from P140 SOD1^G93A^/LAG-3^+/+^ and SOD1^G93A^/LAG-3^−/−^ mice. Principal component analysis showed clear separation between the two genotypes along PC1 (43.3%; Fig. 6C), indicating substantial transcriptional differences. Gene Ontology analysis of biological processes revealed that genes upregulated in SOD1^G93A^/LAG-3^−/−^ spinal cord were enriched in oxidative phosphorylation, energy metabolism, and cellular respiration pathways (Fig. 6D). Conversely, genes downregulated in SOD1^G93A^/LAG-3^−/−^ spinal cord were enriched in inflammatory and immune response pathways, including activation of the innate immune response, regulation of cytokine production, and interleukin-related signaling (Fig. 6E).

Together, these results demonstrate that LAG-3 deletion enhances *in vivo* microglial lysosomal activity and ameliorates the spinal cord transcriptional environment at the late disease stage, with suppression of inflammatory and immune pathways alongside restoration of energy metabolism gene expression.

## Discussion

In this study, we investigated the role of LAG-3 in microglial regulation during ALS pathogenesis using SOD1^G93A^ mice. Our findings demonstrate that LAG-3 is progressively upregulated in spinal cord microglia, that LAG-3-high microglia exhibit a DAM transcriptional signature, and that genetic deletion of LAG-3 produces a biphasic phenotype: accelerated disease onset but prolonged disease duration. Mechanistically, LAG-3 deficiency dissociates the inflammatory and phagocytic modules within the DAM program in a stage-dependent manner. This dissociation translates into enhanced microglial phagocytic capacity and amelioration of the spinal cord transcriptional environment at the late disease stage. These findings demonstrate that LAG-3 is a stage-dependent regulator of microglial function in ALS.

We observed progressive upregulation of LAG-3 in the spinal cord of SOD1^G93A^ mice, with immunohistochemical analysis confirming its predominant localization to Iba1^+^ microglia/macrophages (Fig. 1). This microglial expression is consistent with multiple independent reports [31–33] and contrasts with the initial proposal that LAG-3 mediates neuronal α-synuclein uptake [34], which has not been supported by subsequent studies [35]. We previously demonstrated that IFN-γ induces LAG-3 expression in microglia [26], and IFN-γ is known to be elevated in the ALS spinal cord, produced by infiltrating T cells and activated microglia [36, 37]. The lack of LAG-3 elevation in peripheral lymphoid organs (Sup. Fig. 1A–C) indicates that this response is CNS-intrinsic. Notably, LAG-3 expression was regionally restricted to the ventral horn and intermediate zone, where motor neuron degeneration occurs, with minimal expression in the dorsal horn (Sup. Fig. 1F). This regional pattern probably also explains the marked heterogeneity in LAG-3 expression among microglia within the same spinal cord: microglia residing in close proximity to degenerating motor neurons are likely exposed to higher local concentrations of LAG-3-inducing signals such as IFN-γ than those farther from active pathology. Whether the stable maintenance of the LAG-3-high state also involves epigenetic mechanisms is an important question for future investigation.

The DAM transcriptional program is now established across neurodegenerative diseases including ALS [13, 15, 18, 19], yet the molecular signals that drive individual microglia toward this state have remained incompletely understood. Our transcriptomic profiling of FACS-sorted LAG-3-high versus LAG-3-low microglia from SOD1^G93A^ spinal cord revealed that LAG-3-high microglia exhibit a canonical DAM signature, characterized by upregulation of lysosomal, efferocytosis, and cholesterol metabolism pathways, and downregulation of cell cycle-related genes (Fig. 2F, G), with the gradual transition from WT through LAG-3-low to LAG-3-high populations corresponding to progressive DAM transformation (Fig. 2E). Immunohistochemical analysis further confirmed that LAG-3-high Iba1^+^ cells display elevated CD68 immunoreactivity and an activated, amoeboid-like morphology (Sup. Fig. 2C–G), demonstrating that the DAM signature is recapitulated at both the protein and morphological levels. These observations raise the question of whether LAG-3 is a passive marker of the DAM state or an active regulator of the homeostatic-to-DAM transition. Our data from LAG-3-deficient mice support an active regulatory role: genetic deletion of LAG-3 prevented the full transcriptional shift toward the DAM state at the late disease stage, with LAG-3 knockout microglia clustering closer to the early-stage transcriptional profile rather than progressing to the characteristic late-stage DAM pattern (Fig. 4A-C). This finding indicates that LAG-3 is required for the execution of the DAM program in ALS pathology.

The biphasic phenotype of SOD1^G93A^/LAG-3^−/−^ mice, accelerated onset combined with prolonged disease duration, initially appears to contradict our previous demonstration that LAG-3 knockdown enhances acute inflammatory responses in microglia [26]. This apparent discrepancy resolves once the temporal dimension of LAG-3 function is considered. Our previous study examined the acute response of LAG-3-deficient microglia to short-term inflammatory stimulation in vitro, in which LAG-3 functioned as a brake on inflammatory output. The early-stage phenotype of SOD1^G93A^/LAG-3^−/−^ mice in the present study, upregulation of inflammatory pathways at P120 (Fig. 4E) and accelerated disease onset (Fig. 3A, B), is consistent with this anti-inflammatory brake function. By P140, however, SOD1^G93A^ microglia have undergone weeks of chronic activation and have progressed deep into the DAM transcriptional state, which involves co-activation of inflammatory and phagocytic effector genes. In this chronic context, LAG-3 no longer influences transient inflammatory responses but helps sustain the DAM state where both modules are coupled. Constitutive LAG-3 deficiency allows microglia to bypass entry into this fully coupled DAM state, as evidenced by their transcriptional resemblance to the early-stage profile at P140 (Fig. 4A) and by the selective preservation of phagocytic effector genes (*Axl, Itgax*) alongside suppression of inflammatory DAM components (*Clec7a, Apoe*; Fig. 4F, G). The inflammatory and phagocytic modules within the DAM program are therefore dissociable, and LAG-3 functions stage-dependently: as a brake on acute inflammatory responses early in disease, and as a maintainer of a coupled inflammatory–phagocytic DAM state in the chronic phase.

Functional validation confirmed that this transcriptional reprogramming has cellular and tissue-level consequences. Primary microglia from late-stage LAG-3-deficient mice exhibited enhanced phagocytic uptake (Fig. 5A-C), and re-introducing LAG-3 into these cells reduced their phagocytic capacity (Fig. 5D, E), demonstrating that LAG-3 cell-autonomously restrains phagocytic activity. Bulk RNA-seq of the P140 spinal cord further revealed coordinated suppression of inflammatory and innate immune pathways alongside restoration of oxidative phosphorylation and energy metabolism gene expression (Fig. 6C-E). Chronic neuroinflammation is a recognized driver of disease progression in ALS [4, 5], and mitochondrial dysfunction is a well-known pathological hallmark of the ALS spinal cord [38–40]. The concurrent suppression of inflammation and restoration of OXPHOS-related gene expression provides a mechanistic basis for the prolonged disease duration observed in SOD1^G93A^/LAG-3^−/−^ mice.

Our findings, together with recent work on TIM-3 in Alzheimer’s disease [23], begin to reveal a broader role for immune checkpoint molecules in microglial regulation during neurodegeneration. The two checkpoint molecules exert opposing effects on DAM trajectory: TIM-3 deletion promotes DAM transition, whereas LAG-3 deletion inhibits it. Despite this directional difference, both molecules converge on a shared functional outcome: dissociation of the phagocytic and inflammatory modules, with enhanced phagocytic gene expression and suppressed proinflammatory signaling. This convergence suggests that immune checkpoint molecules do not regulate the DAM program as a monolithic entity but rather act on its individual functional modules, and that distinct checkpoint molecules may be leveraged to selectively modulate specific DAM components. From a therapeutic perspective, this module-level regulation is encouraging: it suggests that temporally controlled inhibition of specific checkpoint molecules could enhance beneficial microglial functions such as phagocytosis without exacerbating neuroinflammation, provided the intervention is calibrated to the disease stage at which each checkpoint’s dominant function shifts.

Several limitations of this study should be noted. First, we used germline LAG-3 knockout mice, which lack LAG-3 throughout development and in all cell types. Although flow cytometric profiling identified microglia as the dominant LAG-3-expressing immune cell type within the SOD1^G93A^ spinal cord (Sup. Fig. 1D, E), and our rescue experiment demonstrated cell-autonomous LAG-3 function in microglia (Fig. 5D, E), contributions from LAG-3 expressed at lower levels by infiltrating T cells cannot be fully excluded. Microglia-specific conditional knockout models will be necessary to determine the microglia-intrinsic contributions of LAG-3 to disease progression. Second, the effect of LAG-3 deficiency on disease progression was characterized by an approximately 10% extension of disease duration, and demonstrating statistical significance required cohorts of more than 20 mice per group. Finally, the molecular mechanisms underlying LAG-3-mediated regulation of microglial function remain unknown. Identifying LAG-3's microglia-specific signaling cascade and ligands will be essential for understanding how this checkpoint molecule orchestrates the functional balance between inflammatory and phagocytic programs.

## Conclusions

We identify LAG-3 as a stage-dependent regulator of microglial function in ALS that dissociates the inflammatory and phagocytic modules within the DAM program. These findings reveal that the functional output of microglial activation is not determined solely by the activation state itself but is shaped by the regulatory checkpoint present at each disease stage. Our results support the concept that immune checkpoint molecules constitute a class of module-level regulators of microglial function, and that understanding their stage-specific roles will be essential for deciphering the mechanisms underlying microglial dysfunction in neurodegeneration.

## Supplementary Information

Supplementary Material 1: Figure S1: Expression profiling of LAG-3 across peripheral immune tissues and spinal cord immune cell populations in SOD1^G93A^ mice. (A-C) LAG-3 mRNA levels in cervical lymph nodes (A), lumbar lymph nodes (B), and spleen (C). n=3 per group. Mean ± SEM. (D and E) Flow cytometric profiling and quantification of LAG-3 expression across various immune cell populations isolated from the spinal cord of P140 SOD1^G93A^ mice. (D) Representative flow cytometry plots showing the LAG-3 expression levels in microglia and infiltrating peripheral immune cell subsets, including CD8^+^ T cells, CD25^-^/CD4^+^ T cells, CD25^+^/CD4^+^ regulatory T cells (Tregs), and NK cells. (E) Quantitative comparison of LAG-3 expression (Mean Fluorescence Intensity, MFI) among the indicated immune cell populations. n=4 per group. Mean ± SEM. ****p < 0.0001 vs microglia by one-way ANOVA with Dunnett's multiple comparisons test. (F) Low-magnification representative immunohistochemical images for spatial distribution of LAG-3-expressing microglia/macrophages in the spinal cord tissue. Scale bars: 200 μm.

Supplementary Material 2: Figure S2: Phenotypic characterization and transcriptomic functional enrichment analysis of LAG-3-high microglia. (A) Representative flow cytometry plots to isolate or analyze microglial populations from the spinal cord of mice based on CD11b and CD45 expression. The microglial fraction is identified and gated as the CD11b^+^/CD45^int^ population. (B) Quantitative validation of *Havcr2* transcript levels in sorted microglial subpopulations. Mean ± SEM. *p < 0.05 by one-way ANOVA followed by Tukey's post-hoc test. (C-G) Quantitative immunohistochemical validation of the lysosomal DAM marker CD68 and morphological analysis in LAG-3-defined microglial subpopulations within P140 SOD1^G93A^ spinal cord tissue. (C) Representative immunofluorescence images showing CD68 and LAG-3 expression with Iba1^+^ cells. Scale bars: 20 μm. (D) Quantification of CD68 immunoreactivity between LAG-3-low and LAG-3-high Iba1^+^ populations. (E) Quantitative analysis of microglial soma size. (F) Quantitative analysis of microglial cell circularity. (G) Quantitative analysis of microglial cell solidity. n=58 cells from 3 mice per group. ***p < 0.001, ****p < 0.0001 by Student's t-test. (H) KEGG pathway enrichment analysis of genes upregulated in LAG-3-high microglia compared to wild-type microglia. (I), (J) Gene Ontology (GO) enrichment analysis of biological processes for genes upregulated (I) and downregulated (J) in LAG-3-high microglia compared to wild-type microglia.

Supplementary Material 3: Figure S3: Evaluation of Dectin-1 levels and stage-dependent astrocytic activation across different LAG-3 genotypes. (A) Representative Western blot analysis of Dectin-1 in the lumbar spinal cord of wild-type (WT), SOD1^G93A^/LAG-3^+/+^, and SOD1^G93A^/LAG-3^-/-^ mice at P140. (B) Densitometric quantification of Dectin-1 protein expression levels. n=3 per group. Mean ± SEM. **p < 0.01 by Student's t-test (SOD1^G93A^/LAG-3^+/+^ vs SOD1^G93A^/LAG-3^-/-^). Dectin-1 was not detected in WT samples. (C) Representative immunofluorescence images of GFAP in lumbar spinal cord across genotypes and disease stages. Scale bars: 200 μm. (D) Quantification of GFAP^+^ area in lumbar spinal cord across genotypes and disease stages. n = 3 mice per genotype at P60 and P100; at P120 and P140, n = 6 mice for SOD1^G93A^/LAG-3^+/+^ and SOD1^G93A^/LAG-3^-/-^, and n = 5 mice for SOD1^G93A^/LAG-3^+/-^. Mean ± SEM.

Supplementary Material 4: Figure S4: Stage-dependent transcriptional reprogramming in LAG-3-deficient microglia.(A) Heatmap displaying z-score normalized expression of homeostatic and DAM markers across four conditions. (B),(C) KEGG pathway enrichment analysis of genes upregulated (B) or downregulated (C) in P140 SOD1^G93A^/LAG-3^-/-^ microglia compared to P140 SOD1^G93A^/LAG-3^+/+^.

Supplementary Material 5: Table S1: Primer sequences.

Supplementary Material 6: Table S2: Detailed information on RNA-seq samples.

## Data Availability

The RNA-sequencing data generated in this study have been deposited in the NCBI Gene Expression Omnibus (GEO) under accession number GSE320562. All other data supporting the findings of this study are available within the article and its supplementary materials.

## Abbreviations

ALS: Amyotrophic lateral sclerosis
BCA: Bicinchoninic acid
CNS: Central nervous system
DAM: Disease-associated microglia
DEG: Differentially expressed gene
DMEM: Dulbecco’s modified Eagle medium
FACS: Fluorescence-activated cell sorting
FBS: Fetal bovine serum
FMO: Fluorescence minus one
GFAP: Glial fibrillary acidic protein
GM-CSF: Granulocyte–macrophage colony-stimulating factor
GO: Gene Ontology
HBSS: Hanks’ balanced salt solution
Iba1: Ionized calcium-binding adapter molecule 1
IFN-γ: Interferon-gamma
KEGG: Kyoto Encyclopedia of Genes and Genomes
KO: Knockout
LAG-3: Lymphocyte activation gene-3
LAMP1: Lysosomal associated membrane protein 1
M-CSF: Macrophage colony-stimulating factor
MFI: Mean fluorescence intensity
OCT: Optimal cutting temperature
OXPHOS: Oxidative phosphorylation
PBS: Phosphate-buffered saline
PCA: Principal component analysis
PCR: Polymerase chain reaction
PFA: Paraformaldehyde
PVDF: Polyvinylidene difluoride
qPCR: Quantitative PCR
RNA-seq: RNA sequencing
SDS-PAGE: Sodium dodecyl sulfate–polyacrylamide gel electrophoresis
SEM: Standard error of the mean
SOD1: Cu/Zn superoxide dismutase 1
TBS: Tris-buffered saline
TIM-3: T-cell immunoglobulin and mucin-domain containing-3
WT: Wild-type
