# Supplementary figures and images for: Immune checkpoint LAG-3 governs stage-dependent and disease-associated microglial modules in ALS model mice
Source: J Neuroinflammation. 2026 Jun 25;23:260. doi: 10.1186/s12974-026-03919-8 (PMC13428418; doi:10.1186/s12974-026-03919-8)

**A**

cervical lymphnode

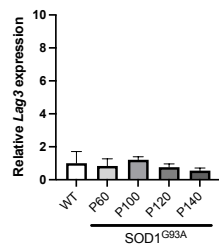**B**

lumbar lymphnode

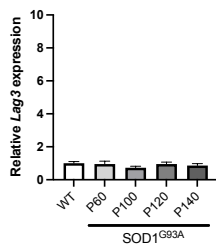**C**

spleen

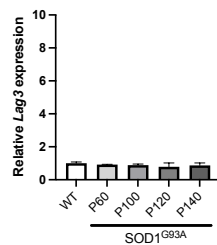**D**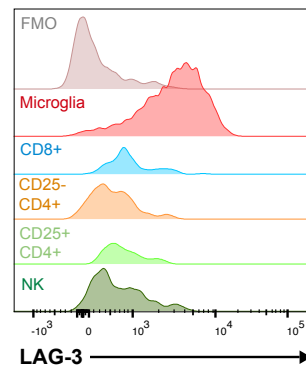**E**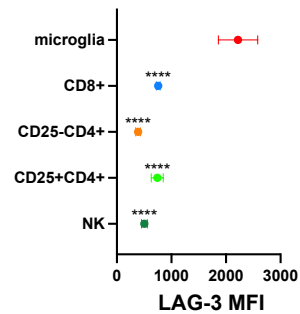**F**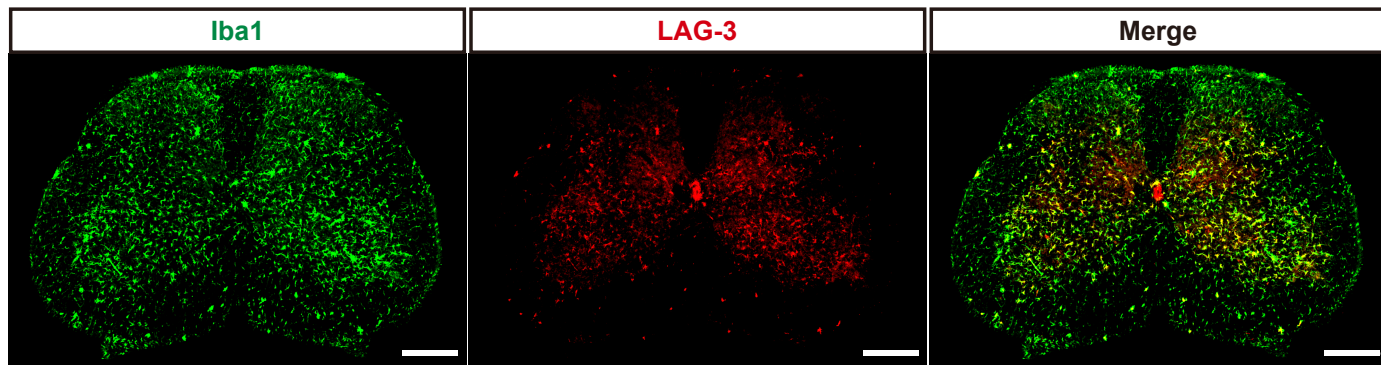

Supplement: Supplementary file 1 — Supplementary Material 1: Figure S1: Expression profiling of LAG-3 across peripheral immune tissues and spinal cord immune cell populations in SOD1G93A mice. (A-C) LAG-3 mRNA levels in cervical lymph nodes (A), lumbar lymph nodes (B), and spleen (C). n=3 per group. Mean ± SEM. (D and E) Flow cytometric profiling and quantification of LAG-3 expression across various immune cell populations isolated from the spinal cord of P140 SOD1G93A mice. (D) Representative flow cytometry plots showing the LAG-3 expression levels in microglia and infiltrating peripheral immune cell subsets, including CD8+ T cells, CD25-/CD4+ T cells, CD25+/CD4+ regulatory T cells (Tregs), and NK cells. (E) Quantitative comparison of LAG-3 expression (Mean Fluorescence Intensity, MFI) among the indicated immune cell populations. n=4 per group. Mean ± SEM. ****p < 0.0001 vs microglia by one-way ANOVA with Dunnett's multiple comparisons test. (F) Low-magnification representative immunohistochemical images for spatial distribution of LAG-3-expressing microglia/macrophages in the spinal cord tissue. Scale bars: 200 μm. [file 12974_2026_3919_MOESM1_ESM.pdf]

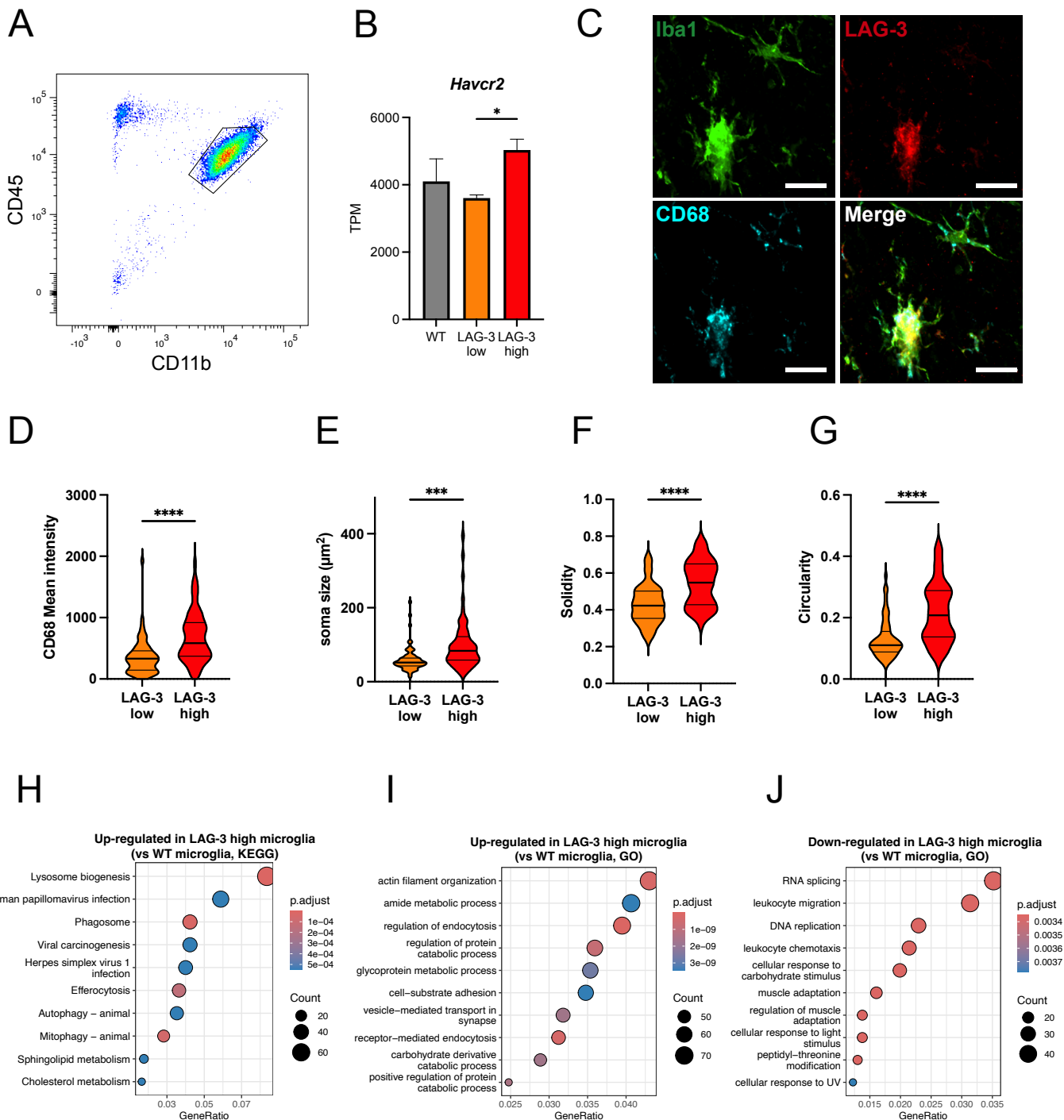

Supplement: Supplementary file 2 — Supplementary Material 2: Figure S2: Phenotypic characterization and transcriptomic functional enrichment analysis of LAG-3-high microglia. (A) Representative flow cytometry plots to isolate or analyze microglial populations from the spinal cord of mice based on CD11b and CD45 expression. The microglial fraction is identified and gated as the CD11b+/CD45int population. (B) Quantitative validation of Havcr2 transcript levels in sorted microglial subpopulations. Mean ± SEM. *p < 0.05 by one-way ANOVA followed by Tukey's post-hoc test. (C-G) Quantitative immunohistochemical validation of the lysosomal DAM marker CD68 and morphological analysis in LAG-3-defined microglial subpopulations within P140 SOD1G93A spinal cord tissue. (C) Representative immunofluorescence images showing CD68 and LAG-3 expression with Iba1+ cells. Scale bars: 20 μm. (D) Quantification of CD68 immunoreactivity between LAG-3-low and LAG-3-high Iba1+ populations. (E) Quantitative analysis of microglial soma size. (F) Quantitative analysis of microglial cell circularity. (G) Quantitative analysis of microglial cell solidity. n=58 cells from 3 mice per group. ***p < 0.001, ****p < 0.0001 by Student's t-test. (H) KEGG pathway enrichment analysis of genes upregulated in LAG-3-high microglia compared to wild-type microglia. (I), (J) Gene Ontology (GO) enrichment analysis of biological processes for genes upregulated (I) and downregulated (J) in LAG-3-high microglia compared to wild-type microglia. [file 12974_2026_3919_MOESM2_ESM.pdf]

A

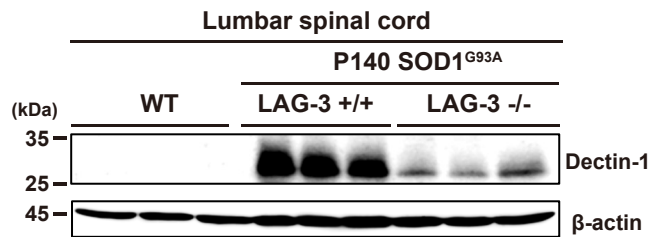

B

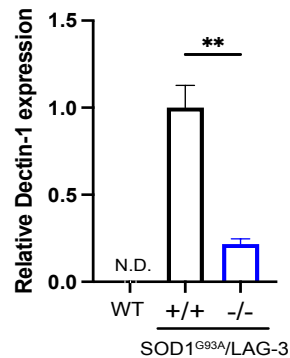

C

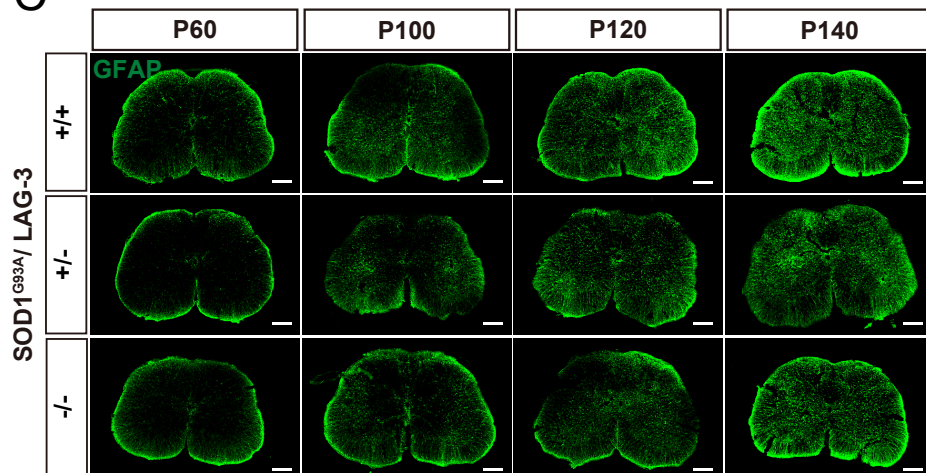

D

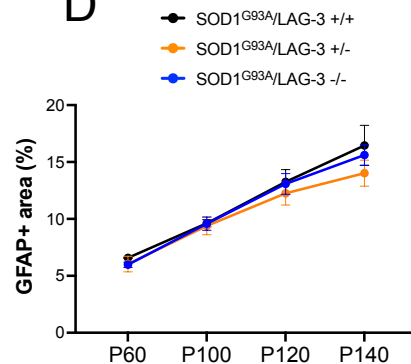

Supplement: Supplementary file 3 — Supplementary Material 3: Figure S3: Evaluation of Dectin-1 levels and stage-dependent astrocytic activation across different LAG-3 genotypes. (A) Representative Western blot analysis of Dectin-1 in the lumbar spinal cord of wild-type (WT), SOD1G93A/LAG-3+/+, and SOD1G93A/LAG-3-/- mice at P140. (B) Densitometric quantification of Dectin-1 protein expression levels. n=3 per group. Mean ± SEM. **p < 0.01 by Student's t-test (SOD1G93A/LAG-3+/+ vs SOD1G93A/LAG-3-/-). Dectin-1 was not detected in WT samples. (C) Representative immunofluorescence images of GFAP in lumbar spinal cord across genotypes and disease stages. Scale bars: 200 μm. (D) Quantification of GFAP+ area in lumbar spinal cord across genotypes and disease stages. n = 3 mice per genotype at P60 and P100; at P120 and P140, n = 6 mice for SOD1G93A/LAG-3+/+ and SOD1G93A/LAG-3-/-, and n = 5 mice for SOD1G93A/LAG-3+/-. Mean ± SEM. [file 12974_2026_3919_MOESM3_ESM.pdf]

A

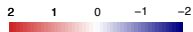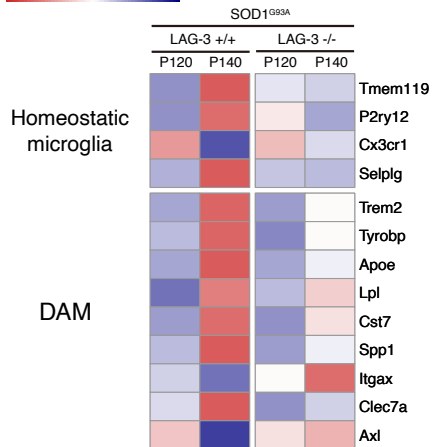

B

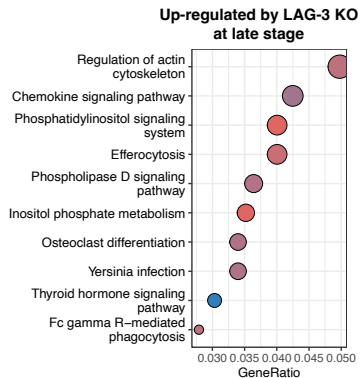

C

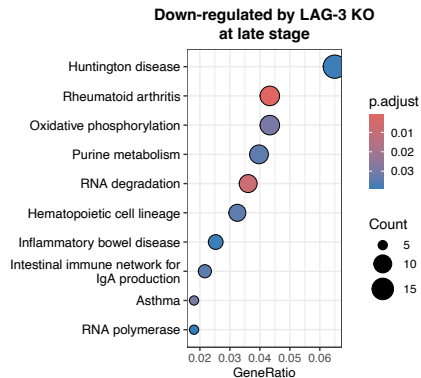

Supplement: Supplementary file 4 — Supplementary Material 4: Figure S4: Stage-dependent transcriptional reprogramming in LAG-3-deficient microglia.(A) Heatmap displaying z-score normalized expression of homeostatic and DAM markers across four conditions. (B),(C) KEGG pathway enrichment analysis of genes upregulated (B) or downregulated (C) in P140 SOD1G93A/LAG-3-/- microglia compared to P140 SOD1G93A/LAG-3+/+. [file 12974_2026_3919_MOESM4_ESM.pdf]
